# COVID-19 Messaging on Social Media for American Indian and Alaska Native Communities: Thematic Analysis of Audience Reach and Web Behavior

**DOI:** 10.2196/38441

**Published:** 2022-11-25

**Authors:** Rose Weeks, Sydney White, Anna-Maria Hartner, Shea Littlepage, Jennifer Wolf, Kristin Masten, Lauren Tingey

**Affiliations:** 1 Center for Indigenous Health Department of International Health Johns Hopkins Bloomberg School of Public Health Baltimore, MD United States; 2 Project Mosaic Denver, CO United States

**Keywords:** COVID-19, American Indian or Alaska Native, social media, communication, tribal organization, community health, infodemiology, Twitter, online behavior, content analysis, thematic analysis

## Abstract

**Background:**

During the COVID-19 pandemic, tribal and health organizations used social media to rapidly disseminate public health guidance highlighting protective behaviors such as masking and vaccination to mitigate the pandemic’s disproportionate burden on American Indian and Alaska Native (AI/AN) communities.

**Objective:**

Seeking to provide guidance for future communication campaigns prioritizing AI/AN audiences, this study aimed to identify Twitter post characteristics associated with higher performance, measured by audience reach (impressions) and web behavior (engagement rate).

**Methods:**

We analyzed Twitter posts published by a campaign by the Johns Hopkins Center for Indigenous Health from July 2020 to June 2021. Qualitative analysis was informed by in-depth interviews with members of a Tribal Advisory Board and thematically organized according to the Health Belief Model. A general linearized model was used to analyze associations between Twitter post themes, impressions, and engagement rates.

**Results:**

The campaign published 162 Twitter messages, which organically generated 425,834 impressions and 6016 engagements. Iterative analysis of these Twitter posts identified 10 unique themes under theory- and culture-related categories of framing knowledge, cultural messaging, normalizing mitigation strategies, and interactive opportunities, which were corroborated by interviews with Tribal Advisory Board members. Statistical analysis of Twitter impressions and engagement rate by theme demonstrated that posts featuring culturally resonant community role models (*P*=.02), promoting web-based events (*P*=.002), and with messaging as part of Twitter Chats (*P*<.001) were likely to generate higher impressions. In the adjusted analysis controlling for the date of posting, only the promotion of web-based events (*P*=.003) and Twitter Chat messaging (*P*=.01) remained significant. Visual, explanatory posts promoting self-efficacy (*P*=.01; *P*=.01) and humorous posts (*P*=.02; *P*=.01) were the most likely to generate high–engagement rates in both the adjusted and unadjusted analysis.

**Conclusions:**

Results from the 1-year Twitter campaign provide lessons to inform organizations designing social media messages to reach and engage AI/AN social media audiences. The use of interactive events, instructional graphics, and Indigenous humor are promising practices to engage community members, potentially opening audiences to receiving important and time-sensitive guidance.

## Introduction

### Background

The COVID-19 pandemic has disproportionately affected American Indian and Alaska Native (AI/AN) peoples from a health, economic, and spiritual perspective. In August 2020, rates of confirmed COVID-19 cases among AI/AN peoples were 3.5 times higher than non-Hispanic White populations [1]. AI/AN peoples are more likely to live in multigenerational households, making social distancing challenging [2]. Further, AI/AN individuals are more likely to have preexisting medical conditions that amplify the risk of severe COVID-19 disease, such as obesity and diabetes [3]. Such health inequities are rooted in hundreds of years of Western aggression, ranging from genocide to forced institutionalization (ie, boarding schools) that removed Indigenous peoples from protective cultural practices and perpetuated continuing oppression and socioeconomic inequities [4-7]. During the COVID-19 pandemic, health systems starved by years of federal underspending were called upon to treat a flood of cases of the novel virus [2,8]. Communities that have come together during hardship in sacred ceremony since time immemorial were urged and often mandated by tribal law to stay home, with traditional wisdom keepers at risk for severe disease [8].

Despite these layered challenges, tribal and urban Indian organizations showed remarkable agility and resilience in initiating and promoting mitigation measures such as curfews and social distancing orders that many adjacent non-AI/AN communities implemented briefly or not at all [8]. By the spring of 2021, when access to COVID-19 vaccines became widespread, uptake among AI/AN peoples was the highest of any US racial group, although there were variations across regions and tribal lands [9]. This high acceptance has been attributed to Indigenous values, including solidarity and respect for elders and other culture-bearers threatened by COVID-19 [10]. Innovative and highly varied approaches in delivering and encouraging vaccination were also successful within AI/AN communities [8-11].

To increase confidence in vaccines and other pandemic mitigation strategies, tribes and AI/AN organizations used culturally tailored messaging strategies. Communication campaigns highlighted cultural strengths such as reverence for elders and community members using slogans such as “Be a Good Relative” and “For the Love of Our People” [10,12]. Such campaigns often used social media to disseminate guidance and foster connectedness. Social media also aided in countering the marginalization and erasure of AI/AN peoples, sometimes omitted as a distinct population in national communications about the pandemic’s effects [12]. Prior to the pandemic, social media had provided a sense of power and control over Indigenous identities [13,14]. Now, forums such as the Facebook groups Social Distance Powwow and American Indian COVID-19 Resources and Response have helped participants celebrate traditional skills such as beading and dancing to cope with pandemic losses during a time of social isolation [15,16].

Pandemic-era communication campaigns targeting AI/AN communities used social media to disseminate guidance, meet community needs, and help people stay connected to protective culture and community [17]. Campaigns used proven public health communication strategies, such as engaging trusted leaders to deliver culturally adapted messaging [18]. However, evidence-based guidance on using social media to raise awareness about public health measures was limited at the start of the COVID-19 pandemic, particularly with regard to AI/AN communities. Social media outreach has substantial benefits, especially in the context of a rapidly evolving pandemic, by allowing for immediacy and the ability to forge rapid connections, build rapport with audiences, and dispel rumors by providing accurate information [19,20]. Limitations include the need to monitor channels for harmful misinformation—for instance, in negative comments, which can influence viewers’ opinions [21,22]. There is some evidence that well-designed social media campaigns may deliver a range of behavior change components, such as social support, observational learning, instructions on how to perform a behavior, and prompts or cues to practice a behavior [23]. Public health campaigns using social media have been linked with public health impact such as increases in human papillomavirus vaccination coverage and uptake of pre-exposure prophylaxis prescriptions [24,25]. To compete in dense and rapidly changing social media environments rife with misinformation, public health organizations must design social media campaigns using best practices and compare evidence about what works to reach and engage web-based audiences with protective health messaging [26].

### Johns Hopkins Center for Indigenous Health Campaign

The Johns Hopkins Center for Indigenous Health (CIH; The Bloomberg School’s Center for American Indian Health was renamed Center for Indigenous Health in September 2022) launched a COVID-19 communications campaign at the start of the pandemic in March 2020 and, over the next several months, established a social media presence to inform and connect tribes, urban Indian organizations, and community members with reliable, culturally adapted communication on evidence-based measures to slow the spread of the virus. CIH convened a Tribal Advisory Board (TAB) and engaged AI/AN colleagues based in Arizona, Maryland, Minnesota, New Mexico, and California to guide the social media campaign [27]. The campaign included hundreds of social media posts consisting of graphics, fact sheets, videos, and slideshows, covering topics including physical distancing, mental health, isolation and quarantine, masking, and vaccination and using a variety of tones and message styles. To acknowledge a collective perception of adversity, some posts used insider humor packaged as colorful memes that could be self-deprecating, satiric, or refreshingly silly; such messages were reviewed by the TAB prior to distribution, ensuring the voice, tone, and terms were appropriate for and understood by AI/AN peoples across the country. All social media materials were made available in Microsoft Word–based toolkits including graphics and supporting captions, available for download at a public resource library [28]. Applying guidance from the TAB, CIH’s social media campaign aimed to frame COVID-19 health information with accessible and engaging content featuring Indigenous illustrations and languages across Facebook, Instagram, and Twitter, which is the focus of this study. Although we could not limit our post reach to AI/AN-identifying audiences, to maximize visibility to AI/AN Twitter users, we mentioned leading national organizational accounts on most posts and used hashtags popular with AI/AN Twitter users.

On Twitter, posts reached increasingly larger audiences throughout the campaign, with some messages organically reaching tens of thousands of people. In November 2020 and May 2021, two Twitter Chats (live, open-discussion, and time-bound Twitter campaign events) were organized around, first, Native American Heritage Month, and second, the rollout of COVID-19 vaccines for people of all ages in the United States. Such events reached a relatively large audience, but other posts shared during the campaign achieved 100 or fewer impressions. The divergence in audience reach and impressions throughout the 1-year campaign demonstrated a need to better characterize the relationship between message characteristics and audience reach and engagement. This analysis aimed to describe the correlation of content themes with Twitter post performance in a health campaign aiming to inform AI/AN communities.

## Methods

### Ethical Considerations

This study was reviewed by the Johns Hopkins University Institutional Review Board, which concluded it was not human subjects research since the study encompassed (1) key informant interviews, involving information from individuals about something other than themselves and disclosing no personal opinions; and (2) secondary data analysis.

### Data Source

To examine trends in social media messaging, our analysis focused on Twitter posts shared by CIH from July 1, 2020, to June 30, 2021—a 1-year campaign. During this time frame, CIH published 162 original campaign-related posts. Twitter analytics data were extracted from CIH’s Twitter account, and the analysis reviewed impressions (the number of times a given tweet is viewed); engagements (the number of times a user interacts with a tweet through retweets, favorites, replies, link clicks, hashtag clicks, mention clicks, and media views); and a summary indicator of engagement rate (ER), which measures the number of engagements a tweet has per impression.

To better understand and contextualize themes across Twitter posts, 10 key informant interviews with members of CIH’s TAB were conducted. The TAB was made up of AI/AN and allied health communication professionals from various regions. Meeting twice monthly, the TAB provided guidance on AI/AN public health priorities, reviewed health communications content produced by CIH, and sought to ensure that campaign content was culturally appropriate and relevant across Indian Country. Thus, their feedback shaped all content produced for the Twitter campaign analyzed in this paper.

Key informant interviews with TAB members were conducted by a member of the study team as part of a separate evaluation of the TAB procedures using a semistructured interview guide. Participants were female professionals serving in communications and outreach roles for tribal nations and other organizations serving AI/AN peoples, representing 12 tribes and 10 tribal-serving organizations across various regions; all but 2 identified as AI/AN. Interviews were audio recorded, transcribed, and analyzed by a member of the study team familiar with the data. Although interviews largely focused on participants’ experience in the TAB, passages related to social media strategy were compiled and applied to inform our analysis of Twitter posts.

To guide the analysis and interpretation of results, we used the Health Belief Model (HBM), a theory adapted to influence health behaviors that has been used in diverse cultural contexts since the 1950s [29]. The theory’s 6 constructs include risk susceptibility, risk severity, benefits to action, barriers to action, self-efficacy, and cues to action; the campaign messages aimed to leverage nearly all components [30]. In addition to themes from TAB interviews, risk communication guidance emphasizing the benefits of 2-way communication through social media was also applied to content analysis [19].

### Thematic Coding

Twitter posts were iteratively coded by theme using both deductive content derived from the HBM and TAB interviews and inductive codes based on emergent themes. Thematic coding classified all Twitter posts into categories of similar messaging strategies. In this study, 2 members of the study team each independently coded a subset of 50 posts, initially using deductive themes and then creating additional inductive codes as appropriate. Codes were iteratively refined and combined to create overarching categories through discussion. After 3 revisions, the codebook was finalized, and 2 members of the study team coded all posts, resolving each discrepancy through reflexive discussion. These themes were then used as variables in the analysis of audience reach and engagement.

### Data Cleaning, Exploration, and Analysis in R Statistical Software

Data obtained from CIH’s Twitter account were first entered into R statistical software (version 4.0.3; R Foundation for Statistical Computing) [31]. An initial data exploration stage included data cleaning, in which variables were recategorized and examined for missingness. Data exploration was completed for several variables, including partner tagging, time of day, year and month, and type of post. All date-time variables were parsed to include only the month and year. “High” and “low” ER or impressions were classified through percentiles, in which all posts in the 75th percentile or above in either outcome were classified as “high” and those below the 75th percentile were classified as “low.” Initial descriptive statistics and figures were then used to examine the counts and distribution of posts across the variables of interest using the *dplyr* and *ggplot* packages [32,33]. Possible confounders and a priori variables were evaluated and selected for further analysis. Odds ratios were then calculated to examine the association between theme and impressions or ER; a generalized linear model was used to calculate the adjusted odds ratios. The odds ratios were adjusted for time of year (month and year), which was an a priori variable, to account for several factors over the year-long campaign: an increase in followers over time, a gradual increase in impressions per post, and a decline in average ER per post. Tweets of a particular type where n=1 were excluded from the analysis.

## Results

### Thematic Analysis

The process of coding Twitter posts led to 4 overarching categories, as seen in Multimedia Appendix 1. Of the 162 tweets, 75 (46.3%) were categorically coded as *Framing Knowledge*, 37 (22.8%) as *Cultural Messaging*, 24 (14.8%) as *Normalizing COVID Mitigation Strategies*, and 26 (16%) as *Interactive Opportunities*. Under these 4 categories, the data revealed 10 themes: *Perceived Susceptibility, Perceived Severity, Perceived Benefits, Self-Efficacy, Indigenous Value Systems, Humor, Social Norms, Observational Learning, Event Promotion,* and *Twitter Chat* (see Multimedia Appendix 1). Definitions and examples of tweets coded within each category and theme are shown in Multimedia Appendix 1.

### Engagement and Reach Analysis by Theme

**Figure 1:**
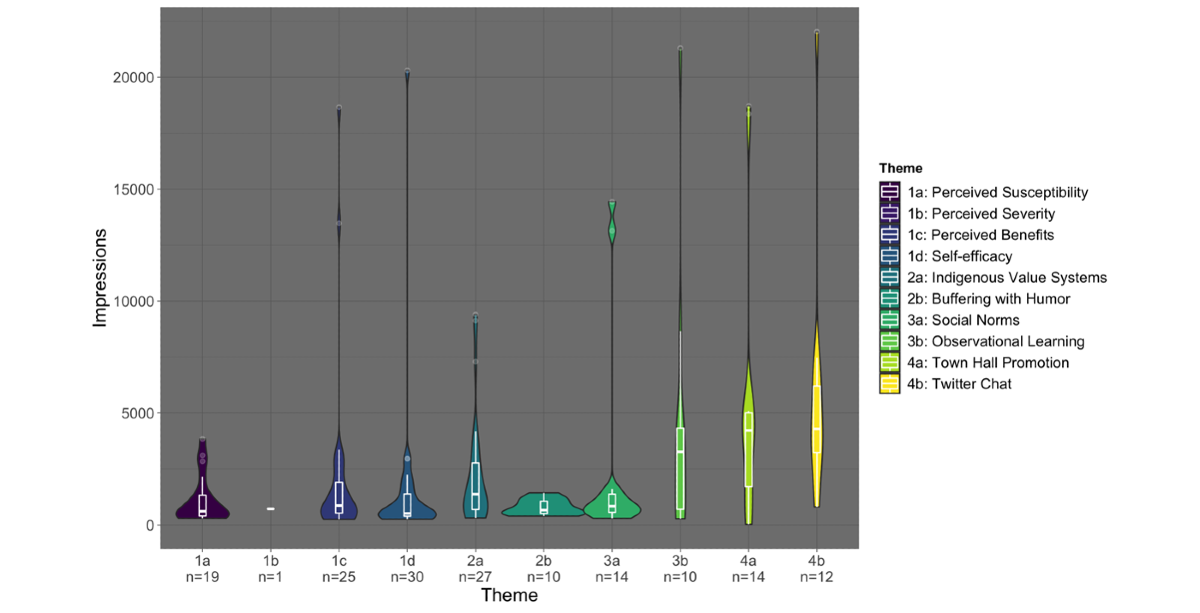
Tweet impressions by social media post theme classification (n=162).

**Figure 2:**
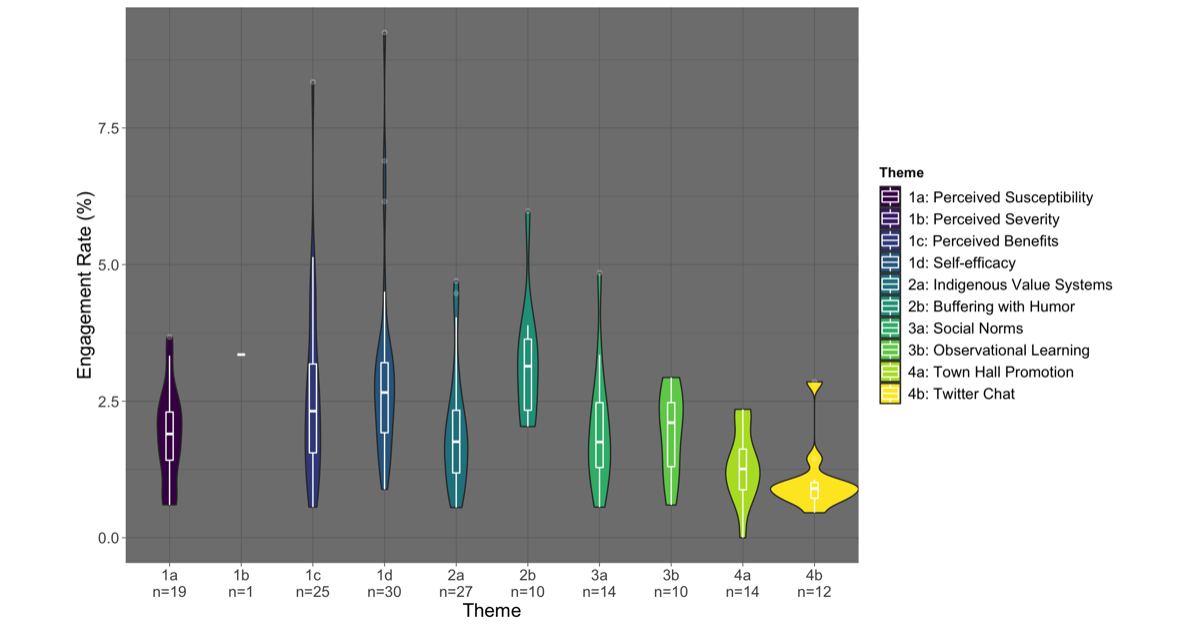
Tweet engagement rate by social media post theme classification (n=162).

CIH’s Twitter account had 900 followers as of February 23, 2021—near the middle of the campaign—which increased to 1200 followers by its end. Throughout the campaign, posts organically generated 425,834 impressions and 6016 engagements. On average, each post received 2628 impressions and 37 engagements, with an average ER of 2.2%. Figures 1 and 2 display the distribution of impressions and ER by the post theme, highlighting initial summary statistics and the density of the distribution. In our data exploration phase, several variables—partner tagging, time of day, and type of post—were not found to be significant and were thus excluded from consideration in the adjusted analysis.

### Post Themes and Impressions

**Figure 3:**
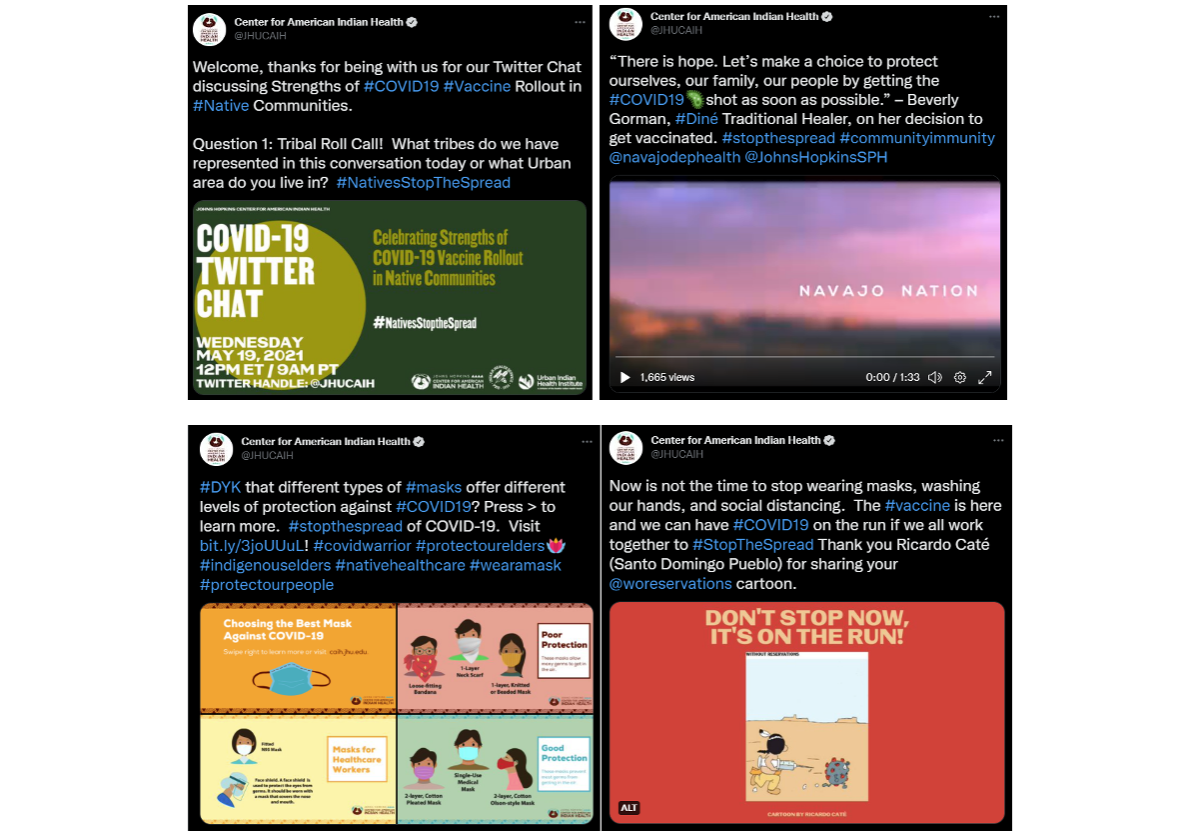
Clockwise from top left: the top-ranked post by impressions was coded as Twitter Chat; the second highest post by impressions was coded as Observational Learning; a top-ER post showing a cartoon Native American individual chasing a COVID-19 particle with a vaccine syringe was coded as Humor; and the highest ranked post by ER was coded as Self-Efficacy. ER: engagement rate.

Table 1 describes post themes and associations with impressions, before and after an adjustment for the date the posts were published. Prior to adjusting for month of posting, posts coded with the theme *Observational Learning* had 5.01 (95% CI 1.36-20.57) times the odds of achieving high impressions than other posts. After adjusting, *Event Promotion* posts had 6.79 (95% CI 1.75-32.27) times the odds of being among the top 75% of tweets by impressions than other posts, and *Twitter Chat* messages had 15.94 (95% CI 3.12-138.42) times the odds of achieving high impressions. The post with the greatest number of impressions (n=22,039) was a *Twitter Chat* welcome message about COVID-19 vaccinations in AI/AN communities (Figure 3). The post with the second highest number of impressions (n=21,309) featured a video with a Navajo traditional healer speaking about her decision to get vaccinated against COVID-19, coded as *Observational Learning* (Figure 3)*.* The reverse trend was observed for posts that were coded as *Self-Efficacy* and *Social Norms,* with these posts having 0.04 (95% CI 0.002-0.31) times and 0.17 (95% CI 0.002-0.73) times the odds of achieving high impressions than other themes.

**Table 1:** Unadjusted and adjusted odds of high impressions by theme.

Theme	Unadjusted OR^a^ (95% CI)	Unadjusted *P* value^b^	Adjusted OR (95% CI)^c^	Adjusted *P* value^b^
1a. Perceived Susceptibility	0.31 (0.05-1.16)	.13	0.70 (0.09-3.70)	.70
1b. Perceived Severity	N/P^d^	N/A^e^	N/P	N/A
1c. Perceived Benefits	0.51 (0.14-1.46)	.35	0.57 (0.14-1.95)	.40
1d. Self-efficacy	0.08 (0.004-0.39)	.001	0.04 (0.002-0.31)	.01
2a. Indigenous Value Systems	1.04 (0.38-2.58)	.94	2.85 (0.82-10.38)	.10
2b. Humor	N/P	N/A	N/P	N/A
3a. Social Norms	0.47 (0.07-1.81)	.33	0.17 (0.002-0.73)	.03
3b. Observational Learning	5.01 (1.36-20.57)	.02	3.40 (0.86-15.18)	.09
4a. Event Promotion	6.53 (2.10-22.53)	.002	6.79 (1.75-32.27)	.003
4b. Twitter Chat	19.19 (4.76-129.15)	<.001	15.94 (3.12-138.42)	.01

^a^OR: odds ratio.

^b^Significant level at *P*<.05.

^c^Adjusted for time of year, see methodology for further details.

^d^N/P: not possible, as the small sample size for these categories leads to 0 values that make the values infinite.

^e^N/A: not applicable.

### Post Theme and Engagement Rate

Posts thematically coded under *Self-Efficacy* and *Humor* were statistically more likely to generate a high ER, with *Self-Efficacy* posts having 2.95 (95% CI 1.27-6.84) times and *Humor* posts having 5.43 (95% CI 1.43-20.70) times the odds of being in the high percentile for ER (Table 2). The post with the highest ER (9%) explained how to wear masks to protect against COVID-19, with simple graphics illustrating masks offering poor protection, such as bandanas, and masks offering good protection, such as disposable surgical masks (Figure 3). An example of a high-ER *Humor* post (ER 6%) was an AI/AN-drawn cartoon of a man with a feather chasing a SARS-CoV-2 particle with a giant syringe, under a headline that read, “Don’t Stop Now—It’s on the Run!” (Figure 3). The post themes associated with having a higher number of impressions in our adjusted analysis, *Event Promotions* and *Twitter Chats*, were not more likely to generate higher ER.

**Table 2:** Unadjusted and adjusted odds of high engagement by theme.

Theme	Unadjusted OR^a^ (95% CI)	Unadjusted *P* value^b^	Adjusted OR (95% CI)^c^	Adjusted *P* value^b^
1a. Perceived Susceptibility	0.31 (0.05-1.16)	.13	0.29 (0.06-1.36)	.12
1b. Perceived Severity	N/P^d^	N/A^e^	N/P	N/A
1c. Perceived Benefits	1.85 (0.72-4.51)	.19	1.99 (0.80-4.99)	.14
1d. Self-efficacy	2.84 (1.22-6.55)	.01	2.95 (1.27-6.84)	.01
2a. Indigenous Value Systems	0.63 (0.20-1.66)	.37	0.61 (0.21-1.74)	.35
2b. Humor	5.01 (1.36-20.57)	.02	5.43 (1.43-20.70)	.01
3a. Social Norms	0.47 (0.07-1.81)	.33	0.48 (0.10-2.28)	.36
3b. Observational Learning	0.72 (.11-3.04)	.69	0.74 (0.15-3.67)	.72
4a. Event Promotion	N/P	N/A	N/P	N/A
4b. Twitter Chat	0.25 (0.01-1.35)	.19	0.23 (0.03-1.84)	.17

^a^OR: odds ratio.

^b^Significant level at *P*<.05.

^c^Adjusted for time of year, see methodology for further details.

^d^N/P: not possible, as the small sample size for these categories leads to 0 values that make the values infinite.

^e^N/A: not applicable.

## Discussion

### Principal Findings

This study thematically analyzed Twitter posts from a COVID-19 communications campaign prioritizing AI/AN audiences to understand how best to reach and engage audiences with pandemic mitigation guidance. The study organized posts into 4 categories and 10 themes that integrated the HBM and risk communication guidance with Indigenous cultural values such as solidarity and humor. On average, posts that highlighted interactive opportunities to learn about and discuss pandemic and cultural issues were likely to reach more people but were not associated with higher engagement. Posts highlighting cultural role models such as traditional healers and web-based influencers often reached high numbers of people, although this finding did not remain significant after adjustment. In contrast, posts that highlighted instructional content with simple graphics or used insider humor to convey pandemic-related guidance were more likely to create high ER but, on average, reached fewer accounts.

In all, 99 (61.1%) out of 162 posts were coded with themes from the HBM, demonstrating that this theory was a strong fit for coding posts. An additional 37 (22.8%) posts were coded with cultural themes, appealing to traditional identities that are vital to the well-being of AI/AN peoples, as demonstrated by their protective effect on binge substance use, suicide attempts, and other major health risks [34,35]. Within these cultural themes, 10 posts were thematically coded as *Humor*. Finally, the *Interactive Opportunities* theme highlighted 2-way communication aiming to augment community connectedness and increase public health transparency during an uncertain time [36].

In our statistical analysis of theme by impressions, we found that posts highlighting web-based events were more likely to achieve a higher reach. Twitter Chats have been a successful strategy to build community for other public health organizations, practitioners, and health advocacy groups [37-39]. The high number of impressions for *Twitter Chat* messaging in this campaign could reflect interaction with other AI/AN–serving organizations as these messages were promoted and shared with a larger audience base. Thus, encouraging 2-way communication in our campaign was successful in reaching more users even if individual users were not as likely to engage directly with campaign posts.

In our study, posts highlighting role models (ie, *Observational Learning*) were not associated with high impressions after adjusting for the date of posting. This finding runs in contrast to evidence from a variety of community contexts, including a campaign reaching tribal audiences, that posts sharing personal stories from trusted messengers can successfully engage audiences [24,40-44]. However, given the importance of storytelling in AI/AN communities, we feel that highlighting trusted role models is critical to successful communication campaigns and warrants future implementation and evaluation within AI/AN contexts.

Audience engagement varied substantially during the campaign, with a reported average ER of 2.2% and a median ER of 1.9% during the 1-year campaign. Although engagement metrics vary by social media platform, industry, and topic, an industry source estimates that nonprofit posts on Twitter average an ER of 0.05% [45]. There is very limited evidence related to Twitter campaigns targeting AI/AN community members, but an evaluation of an AI/AN-oriented obesity prevention campaign observed that social media posts “generated little involvement and response,” and a campaign addressing kidney donation found community members did not engage with Twitter messages during the campaign [46,47]. More research is needed to inform the development of social media content to ensure adequate reach and engagement in AI/AN communities across a range of issues.

Thematically, humorous posts and those with instructional graphics were more likely to spur web-based activity. Other evaluations have found that visual concepts positively affect engagement behaviors, including in AI/AN-focused social media campaigns [48,49]. Our finding that humorous posts were more likely to achieve high ER is bolstered by evidence that scientific visualizations in humorous form can improve knowledge acquisition and problem-solving skills [50,51]. Further, humor has special salience in AI/AN communities as a source of resilience to hardship. Individuals have a great deal of institutional mistrust and may use humor in code switching [52,53]. Across a variety of Indigenous cultures worldwide, humor demonstrates cultural understanding, whereas speaking familiarly and using terms recognized to be of Indigenous origin, such as “Stoodis” and “Skoden”—as the CIH campaign did—can build confidence that messages are coming from within the community [54]. The strategic use of humor in social media messaging also builds upon AI/AN oral traditions such as “Trickster” stories [54]. Humor can also be a powerful tool for building trust in health care relationships [55,56]. Humorous social media content, therefore, has an important role in culturally competent communication strategies, especially around sensitive health topics. Using humor may destigmatize disparities, stimulate discussion, and prompt care-seeking where appropriate. Social media campaigns aiming to reach AI/AN audiences should consider using audience-tailored humor to convey empathy and humility while ensuring cultural appropriateness.

Over the course of the 1-year campaign, the average number of impressions generated per post increased and the average ER declined, potentially showing that the larger audiences the campaign reached later were on average less likely to take action to share or amplify a message they saw. This finding may reflect natural tension between impressions and ER, due to their reciprocal relationship; outside of COVID-19, other campaigns have also found a trade-off between impressions and engagement [41]. This finding also may reflect changes in public sentiment over time; as the pandemic became less novel, community members may have felt less urgency to reshare guidance. Increased burnout and COVID-19 fatigue may have reduced engagement with pandemic-related guidance.

Our findings may be instructive for others seeking to promote culturally tailored content for AI/AN audiences on social media. Social media activity should be integrated into multimodel communication campaigns designed to reach all community members, including those on tribal lands where broadband internet limitations persist [57,58]. Increasing access to high-speed internet in rural AI/AN communities will contribute to the increased relevance of social media communication in the future [58,59].

Although there are differences across social media networks, findings should be relatable to campaigns conducted across numerous platforms. For example, campaigns on Facebook or Instagram incorporating humorous content and highly visual, instructional guidance may be successful in achieving higher performance metrics than those using other types of content, such as posts explaining the benefits of public health measures, which may seem too conventional to engage savvy web-based audiences. This analysis focused on Twitter as the most consistent CIH social media platform throughout the pandemic. Campaign performance metrics on Facebook were highly variable during the time period and thus differences in reach and engagement may not be attributable to the salience of particular thematic content with our audience [60,61]. Meanwhile, CIH’s Instagram account, being newly established, saw lower performance measures than on Facebook or Twitter. Future research should focus on whether Twitter-based findings remain consistent across other platforms, especially with variation in user characteristics across different platforms [62].

### Limitations

We sought to explore social media metrics during the peak of the COVID-19 pandemic and thus, focused on a limited data set to capture reach and engagement to reflect this unique period of time. Our relatively small data set produced parameters with broad confidence intervals, which limits the strength of the quantitative findings. We integrated only 1 confounding factor in the adjusted analysis, date of posting, and other confounders may be unaccounted for, although other factors we reviewed did not seem to affect performance metrics.

Although the CIH campaign achieved nearly a half-million impressions, the total number of AI/AN peoples in the United States is 9.7 million [63]. The prevalence of social media use among AI/AN peoples is likely similar to that among the general population at around 70% [62,64]. Therefore, the campaign’s Twitter posts did not reach a significant proportion of AI/AN social media users in the United States. Additionally, due to data privacy around social media users, it is impossible to verify that those reached by CIH posts were AI/AN. By engaging and cross-promoting content with prominent AI/AN users and organizations, such as through Twitter Chats, we assume that a large proportion of users reached were AI/AN. However, this is a limitation inherent to all studies using social media analytics data for publicly targeted campaigns.

Finally, given the vast diversity across 574 federally recognized tribes and urban AI/AN communities, our findings may not be widely applicable across all AI/AN audiences. The TAB that supervised the development of the campaign and informed this thematic analysis was representative of a variety of tribes and regions but was almost entirely made up of early-to-midcareer professional women. The perspective of other stakeholders such as male leaders may be underrepresented.

### Conclusions

AI/AN communities have been disproportionately affected by the COVID-19 pandemic. Social media offered a medium to rapidly provide public health guidance and foster cultural connectedness to counteract the isolation and marginalization of Indigenous experiences within the pandemic. Awareness campaigns using social media can benefit from integrating effective strategies to reach and engage increasingly active AI/AN audiences on platforms such as Twitter. In a 1-year social media campaign to disseminate guidance on COVID-19, posts highlighting opportunities for web-based discussion were, on average, likely to reach larger audiences. Humorous tweets and posts with simple, instructional graphics were 2 leading ways to engage audiences by demonstrating humility and promoting confidence in public health guidance as well as encouraging the adoption of preventive behaviors. Further analysis across other social media platforms is needed to inform organizations and tribes seeking to disseminate public health guidance to AI/AN communities.

## Appendix

Multimedia Appendix 1 Table illustrating the thematic coding of tweets with examples.

## Abbreviations

AI/AN: American Indian or Alaska Native
CIH: Johns Hopkins Center for Indigenous Health
ER: engagement rate
HBM: Health Belief Model
TAB: Tribal Advisory Board

## References

[1] (2020). Disparities in COVID-19 illness. Centers for Disease Control and Prevention.

[2] (2020). The National Congress of American Indians calls for more attention to COVID-19 impacts to Indian Country. National Congress of American Indians.

[3] (2019). Disparities. Indian Health Service.

[4] Zephier Olson MD, Dombrowski K (2020). A systematic review of Indian boarding schools and attachment in the context of substance use studies of Native Americans. J Racial Ethn Health Disparities.

[5] Breathett K, Sims M, Gross M, Jackson EA, Jones EJ, Navas-Acien A, Taylor H, Thomas KL, Howard BV, American Heart Association Council on Epidemiology and Prevention, Council on Quality of Care and Outcomes Research, Council on Cardiovascular and Stroke Nursing, Council on Clinical Cardiology, Council on Lifestyle and Cardiometabolic Health (2020). Cardiovascular health in American Indians and Alaska Natives: a scientific statement from the American Heart Association. Circulation.

[6] Godfrey TM, Cordova-Marks FM, Jones D, Melton F, Breathett K (2022). Metabolic syndrome among American Indian and Alaska Native populations: implications for cardiovascular health. Curr Hypertens Rep.

[7] Warne D, Lajimodiere D (2015). American Indian health disparities: psychosocial influences. Soc Personal Psychol Compass.

[8] Doshi S, Jordan A, Kelly K, Solomon D (2020). The COVID-19 response in Indian Country. Center for American Progress.

[9] Hill L, Artiga S (2021). COVID-19 vaccination among American Indian and Alaska Native people. Kaiser Family Foundation.

[10] Haroz EE, Kemp CG, O'Keefe Victoria M, Pocock K, Wilson DR, Christensen L, Walls M, Barlow A, Hammitt L (2022). Nurturing innovation at the roots: the success of COVID-19 vaccination in American Indian and Alaska Native communities. Am J Public Health.

[11] Foxworth R, Redvers N, Moreno MA, Lopez-Carmen VA, Sanchez GR, Shultz JM (2021). COVID-19 vaccination in American Indians and Alaska Natives - lessons from effective community responses. N Engl J Med.

[12] Nagle R (2020). Native Americans being left out of US coronavirus data and labelled as 'other'. The Guardian.

[13] Kral I (2011). Youth media as cultural practice: remote Indigenous youth speaking out loud. Aust Aborig Stud.

[14] Rice ES, Haynes E, Royce P, Thompson SC (2016). Social media and digital technology use among Indigenous young people in Australia: a literature review. Int J Equity Health.

[15] Social Distance Powwow. Facebook.

[16] American Indian COVID-19 Resources & Responses. Facebook.

[17] Kuhn N, Sarkar S, White LA, Hoy J, McCray C, Lefthand-Begay C (2020). Decolonizing risk communication: Indigenous responses to COVID-19 using social media. J Indig Soc Dev.

[18] Lumpkins CY, Goeckner R, Hale J, Lewis C, Gunville J, Gunville R, Daley CM, Daley SM (2022). In our sacred voice - an exploration of tribal and community leader perceptions as health communicators of disease prevention among American Indians in the plains. Health Commun.

[19] (2014). Crisis emergency risk communication: social media and mobile media devices. Centers for Disease Control and Prevention.

[20] Hornmoen H, Backholm K, Hornmoen H, Backholm K (2018). Social media use in crises and risks: an introduction to the collection. Social Media Use in Crisis and Risk Communication.

[21] Choi W, Stvilia B (2015). Web credibility assessment: conceptualization, operationalization, variability, and models. J Assoc Inf Sci Technol.

[22] Kim H, Han JY, Seo Y (2020). Effects of Facebook comments on attitude toward vaccines: the roles of perceived distributions of public opinion and perceived vaccine efficacy. J Health Commun.

[23] Simeon R, Dewidar O, Trawin J, Duench S, Manson H, Pardo Pardo J, Petkovic J, Hatcher Roberts J, Tugwell P, Yoganathan M, Presseau J, Welch V (2020). Behavior change techniques included in reports of social media interventions for promoting health behaviors in adults: content analysis within a systematic review. J Med Internet Res.

[24] Limaye RJ, Holroyd TA, Blunt M, Jamison AF, Sauer M, Weeks R, Wahl B, Christenson K, Smith C, Minchin J, Gellin B (2021). Social media strategies to affect vaccine acceptance: a systematic literature review. Expert Rev Vaccines.

[25] Tesoriero JM, Yuan Y, Newport R, O'Grady T, Cotroneo R, Stevens L, Grisham T, Seo S, Gonzalez C (2022). Assessing the impact of a PrEP Aware Week campaign on PrEP prescription fills in NYS. J Public Health Manag Pract.

[26] (2020). Vaccine misinformation management field guide. United Nations Children’s Fund.

[27] Knowledge center. Tribal Advisory Board Map. Johns Hopkins Center for Indigenous Health.

[28] Resource Library. Johns Hopkins Center for Indigenous Health.

[29] Rosenstock IM, Strecher VJ, Becker MH (1988). Social learning theory and the Health Belief Model. Health Educ Q.

[30] Glanz K, Bishop DB (2010). The role of behavioral science theory in development and implementation of public health interventions. Annu Rev Public Health.

[31] R Core Team (2021). R: a language and environment for statistical computing. R Foundation for Statistical Computing.

[32] Wickham H, François R, Henry L, Müller K (2022). dplyr: a grammar of data manipulation. Tidyverse.

[33] Wickham H (2016). ggplot2: Elegant Graphics for Data Analysis.

[34] Tingey L, Cwik MF, Rosenstock S, Goklish N, Larzelere-Hinton F, Lee A, Suttle R, Alchesay M, Massey K, Barlow A (2016). Risk and protective factors for heavy binge alcohol use among American Indian adolescents utilizing emergency health services. Am J Drug Alcohol Abuse.

[35] Garroutte EM, Goldberg J, Beals J, Herrell R, Manson SM, AI-SUPERPFP Team (2003). Spirituality and attempted suicide among American Indians. Soc Sci Med.

[36] Murthy BB, LeBlanc TT, Vagi SJ, Avchen RN (2021). Going viral: the 3 Rs of social media messaging during public health emergencies. Health Secur.

[37] Rabarison KM, Croston MA, Englar NK, Bish CL, Flynn SM, Johnson CC (2017). Measuring audience engagement for public health Twitter chats: insights from #LiveFitNOLA. JMIR Public Health Surveill.

[38] Thomas TH, Nauth-Shelley K, Thompson MA, Attai DJ, Katz MS, Graham D, Sparacio D, Lizaso C, Utengen A, Dizon DS (2018). The needs of women treated for ovarian cancer: results from a #gyncsm Twitter chat. J Patient Cent Res Rev.

[39] Carroll CL, Bruno K, Ramachandran P (2017). Building community through a #pulmcc Twitter chat to advocate for pulmonary, critical care, and sleep. Chest.

[40] Buffard J, Papasava A (2020). A quantitative study on the impact of emotion on social media engagement and conversion. J Digit Soc Media Mark.

[41] Loft LH, Pedersen EA, Jacobsen SU, Søborg Bolette, Bigaard J (2020). Using Facebook to increase coverage of HPV vaccination among Danish girls: an assessment of a Danish social media campaign. Vaccine.

[42] Ramirez A, Despres C, Chalela P, Weis J, Sukumaran P, Munoz E, McAlister A (2022). Pilot study of peer modeling with psychological inoculation to promote coronavirus vaccination. Health Educ Res.

[43] Dutta-Bergman MJ (2005). Theory and practice in health communication campaigns: a critical interrogation. Health Commun.

[44] Britt Rebecca K, Britt Brian C, Anderson Jenn, Fahrenwald Nancy, Harming Shana (2021). "Sharing Hope and Healing": a culturally tailored social media campaign to promote living kidney donation and transplantation among Native Americans. Health Promot Pract.

[45] Cucu E (2022). 2022 Social media industry benchmarks - know exactly where you stand in your market. Social Insider.

[46] Gittelsohn J, Jock B, Poirier L, Wensel C, Pardilla M, Fleischhacker S, Bleich S, Swartz J, Trude A (2020). Implementation of a multilevel, multicomponent intervention for obesity control in Native American communities (OPREVENT2): challenges and lessons learned. Health Educ Res.

[47] Patten CA, Lando H, Resnicow K, Decker PA, Smith CM, Hanza MM, Burhansstipanov L, Scott M (2018). Developing health communication messaging for a social marketing campaign to reduce tobacco use in pregnancy among Alaska Native women. J Commun Healthc.

[48] Patten CA, Lando H, Resnicow K, Decker PA, Smith CM, Hanza MM, Burhansstipanov L, Scott M (2018). Developing health communication messaging for a social marketing campaign to reduce tobacco use in pregnancy among Alaska Native women. J Commun Healthc.

[49] Schreiner M, Fischer T, Riedl R (2019). Impact of content characteristics and emotion on behavioral engagement in social media: literature review and research agenda. Electron Commer Res.

[50] Su LYF, McKasy M, Cacciatore MA, Yeo SK, DeGrauw AR, Zhang JS (2021). Generating science buzz: an examination of multidimensional engagement with humorous scientific messages on Twitter and Instagram. Sci Commun.

[51] Farinella M (2018). The potential of comics in science communication. J Sci Commun.

[52] Saunkeah Bobby, Beans Julie A, Peercy Michael T, Hiratsuka Vanessa Y, Spicer Paul (2021). Extending research protections to tribal communities. Am J Bioeth.

[53] Guadagnolo BA, Cina K, Helbig P, Molloy K, Reiner M, Cook EF, Petereit DG (2009). Medical mistrust and less satisfaction with health care among Native Americans presenting for cancer treatment. J Health Care Poor Underserved.

[54] Hinzo AM, Clark LS (2019). Digital survivance and Trickster humor: exploring visual and digital Indigenous epistemologies in the #NoDAPL movement. Information, Communication & Society.

[55] Dean RA (2003). Native American humor: implications for transcultural care. J Transcult Nurs.

[56] Kalbfleisch PJ (2009). Effective health communication in Native populations in North America. J Lang Soc Psychol.

[57] Curtis ME, Clingan SE, Guo H, Zhu Y, Mooney LJ, Hser Y (2022). Disparities in digital access among American rural and urban households and implications for telemedicine-based services. J Rural Health.

[58] Duarte ME, Vigil-Hayes M, Zegura E, Belding E, Masara I, Nevarez JC (2021). As a squash plant grows: social textures of sparse internet connectivity in rural and tribal communities. ACM Trans Comput Hum Interact.

[59] Sanders CK, Scanlon E (2021). The digital divide is a human rights issue: advancing social inclusion through social work advocacy. J Hum Rights Soc Work.

[60] Oremus W, Alcantara C, Merrill JB, Galocha A (2021). How Facebook shapes your feed. Washington Post.

[61] Stepanov A (2021). Changes to News Feed in 2021. Meta.

[62] Demographics of social media users and adoption in the United States. Pew Research Center.

[63] Jones N, Marks R, Ramirez R, Ríos-Vargas M (2021). 2020 Census illuminates racial and ethnic composition of the country. United States Census Bureau.

[64] Rushing SC, Stephens D (2011). Use of media technologies by Native American teens and young adults in the Pacific Northwest: exploring their utility for designing culturally appropriate technology-based health interventions. J Prim Prev.

