# Supplementary material for: COVID-19 Messaging on Social Media for American Indian and Alaska Native Communities: Thematic Analysis of Audience Reach and Web Behavior
Source: JMIR Infodemiology. 2022 Nov 25;2(2):e38441. doi: 10.2196/38441 (PMC9709694; doi:10.2196/38441)
Supplement: Multimedia Appendix 1 [file infodemiology_v2i2e38441_app1.docx]

**Multimedia Appendix 1**

Table S1 Thematic Coding of Tweets with Examples

| **Theme Name** | **Definition** | **Example** |
| --- | --- | --- |
| 1. Framing Knowledge | Messages aiming to increase knowledge around COVID-19 and promote behavior change |  |
| 1a. Perceived Susceptibility | Making COVID-19 relevant to Native communities (including discussing COVID risk/health disparities, likelihood of spread, etc.). | A graphic with text and a caption explaining you can spread COVID-19 without knowing you are infected with an illustration of two men hugging, one of whom appears infected. |
| 1b. Perceived Severity | Demonstrating negative effects of COVID-19 (including symptoms/risks) | A graphic with an illustration of an elder woman with a floral mask with text and caption explaining that flu can be dangerous for those over 65. |
| 1c. Perceived Benefits | Demonstrating the importance and effectiveness of mitigation strategies (showing reasons why to utilize mitigation strategies, promoting benefits of mitigation) | A graphic with text and caption saying that a COVID-19 vaccine cannot give you COVID-19, but helps fight off the virus. Features an illustration of a Native healthcare worker. |
| 1d. Self-efficacy | Instructional graphic or link to instructional graphic. Encouraging mitigation strategies and increasing feasibility of strategies by using concrete, instructional content. | A graphic explaining the importance of following social distancing rules by wearing a mask and keeping 6 feet distance when someone in the household is exposed to COVID-19, with an illustration of a man wearing a surgical mask. |
| 2. Cultural Messaging | Emphasizing cultural values supporting COVID-19 prevention approaches to ground guidance. |  |
| 2a. Indigenous Value Systems | Utilizing Indigenous value systems to endorse mitigation strategies or overall community strength to buffer against COVID-19 crisis. | A graphic with text and caption emphasizing the ability to safely stay connected with elders by sharing traditional knowledge virtually with an illustration of a video call between a young woman and an elder woman. |
| 2b. Humor | Approaching COVID-19 and health messaging using humor, an important value among Indigenous people. | A graphic with a photo from the movie *Smoke Signals* and text and caption with a humorous message to get vaccinated, referencing frybread and using Native slang. |
| 3. Normalizing COVID mitigation strategies | Messages that directly target social norms or modeling to increase self-efficacy around COVID-19 mitigation |  |
| 3a. Social Norms | Showcasing vaccination/masks as a community norm across Indian Country. | A graphic showing a young woman learning to drive, featuring text and a caption around learning to watch out for others as you grow up, including through learning to drive and getting vaccinated. |
| 3b. Observational Learning | Spotlighting COVID-19 “heroes,” or those making choices to protect their communities to serve as role models of mitigation behavior. | A graphic with a photo of Two-Spirit Native American Artist Sean Snyder (Navajo/Ute), a caption with their quote discussing Indigenous vaccination, and text wishing a “Happy Pride Month.” |
| 4. Interactive Opportunities | Creating opportunities to discuss concerns and problem-solve. Allows for or promotes real-time opportunities to engage around health issues |  |
| 4a. Town Hall promotion | Promoting interactive webinar activities through invitations to virtual events. | A graphic with registration and promotional information for a webinar series featuring photos of speakers (e.g., Native physician). |
| 4b. Twitter Chat | Inviting real-time participation from partners and users in a collaborative way to discuss health themes in an audience-boosting and impactful space. | A graphic with promotional information around a COVID-19 Twitter chat with a caption posing a question asking for the best campaigns about COVID-19 vaccines. |
| *all codes pertain to COVID-19 directly and “COVID-19 adjacent” topics, including mental health and influenza | | |
